# Inflammatory Papillary Hyperplasia: A Diagnostic Challenge to Oral Physicians

**DOI:** 10.7759/cureus.116291

**Published:** 2026-09-15

**Authors:** Karthik Rajaram Mohan, Ramachandra Reddy Gowda Venkatesha, Saramma Mathew Fenn

**Affiliations:** 1 Oral Medicine and Radiology, Vinayaka Mission's Sankarachariyar Dental College, Vinayaka Mission's Research Foundation (DU), Salem, IND

**Keywords:** carbondioxide laser, cryo ablation, double loop, hard palate, papillary hyperplasia

## Abstract

Inflammatory papillary hyperplasia is a benign, non-neoplastic proliferation of epithelial lining the oral cavity that affects the palatal mucosa with one or more granular, pebbly, nodular, grape, or leaf-like growths. It usually occurs as a soft tissue overgrowth on the hard palate mucosa. It commonly occurs among prolonged or ill-fitting denture wearers, occlusal disharmony resulting from defective occlusal contacts or imbalance between centric relation and occlusion, chronic use of steroid inhalers among asthmatics, immunosuppression like uncontrolled diabetes and AIDS in HIV, poor oral and denture hygiene promote the colonization of candida species and cause underlying mucositis. Rarely, this clinical entity also occurs along the labial aspect of the edentulous mandibular anterior alveolar ridge. Our case discusses a rare clinical presentation of inflammatory papillary hyperplasia in a 49-year-old male who has been a chronic denture wearer for 10 years.

## Introduction

Inflammatory papillary hyperplasia is a clinical condition characterized by numerous pebble-like nodular growths on the denture-bearing surface of the hard palate mucosa [1]. Such growth is painless, non-neoplastic, benign, rare, and encountered in patients wearing dentures for prolonged periods [1]. Smaller lesions appear as areas of intense erythema with multiple elevated granule-like structures resembling granular structures on the oral mucosa [1]. Large lesions exhibit sessile, non-pedunculated pebble-like nodular projections resembling mulberry or grape-like clusters interspersed with non-sessile, pedunculated flat-leaf-like growths [1]. Our case described a varied clinical presentation of inflammatory palatal papillary hyperplasia on the hard palate.

## Case presentation

A 49-year-old man visited our department for a routine dental checkup and screening. He presented with numerous papillary pebble-like projections involving the anterior region of the hard palate. He was a complete maxillary denture wearer both day and night for ten years. He had smoked two bidis a day for two years. Intraoral examination revealed numerous pebble-like elevated nodular growths with diameters ranging from 1 mm to 4 mm deep, localized in the center of the anterior region of the hard palate. Upon inspection, the color of the growth is the same as that of the adjacent mucosa. The growth is surrounded by leaf-like growth that hangs down from the hard palate mucosa and is restricted only to the palatal mucosa tissue-bearing surface of the maxillary denture. On palpation, the growth was non-tender, non-pedunculated, sessile, and firm in consistency except in the lesion's periphery, which showed a leaf-like growth hanging down that is slightly movable (Figure 1).

**Figure 1:**
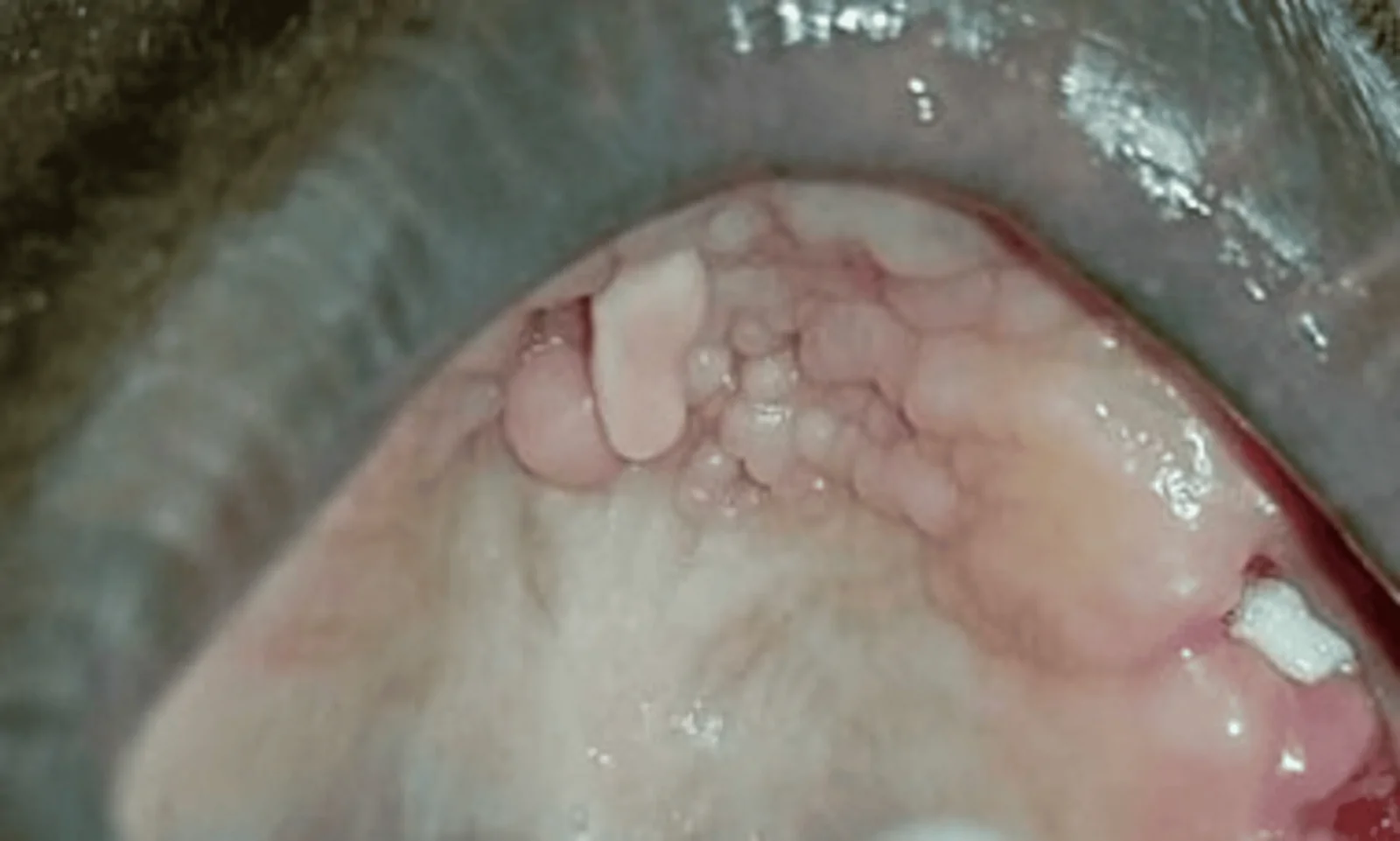
Intraoral clinical examination revealed nodular pebble-like lesions in the center and leaf-like growth near the periphery on the hard palate mucosa

Further intraoral clinical examination revealed a leaf-like growth with a smooth margin hanging down near the mid-palatine raphe region on the hard palate mucosa (Figure 2).

**Figure 2:**
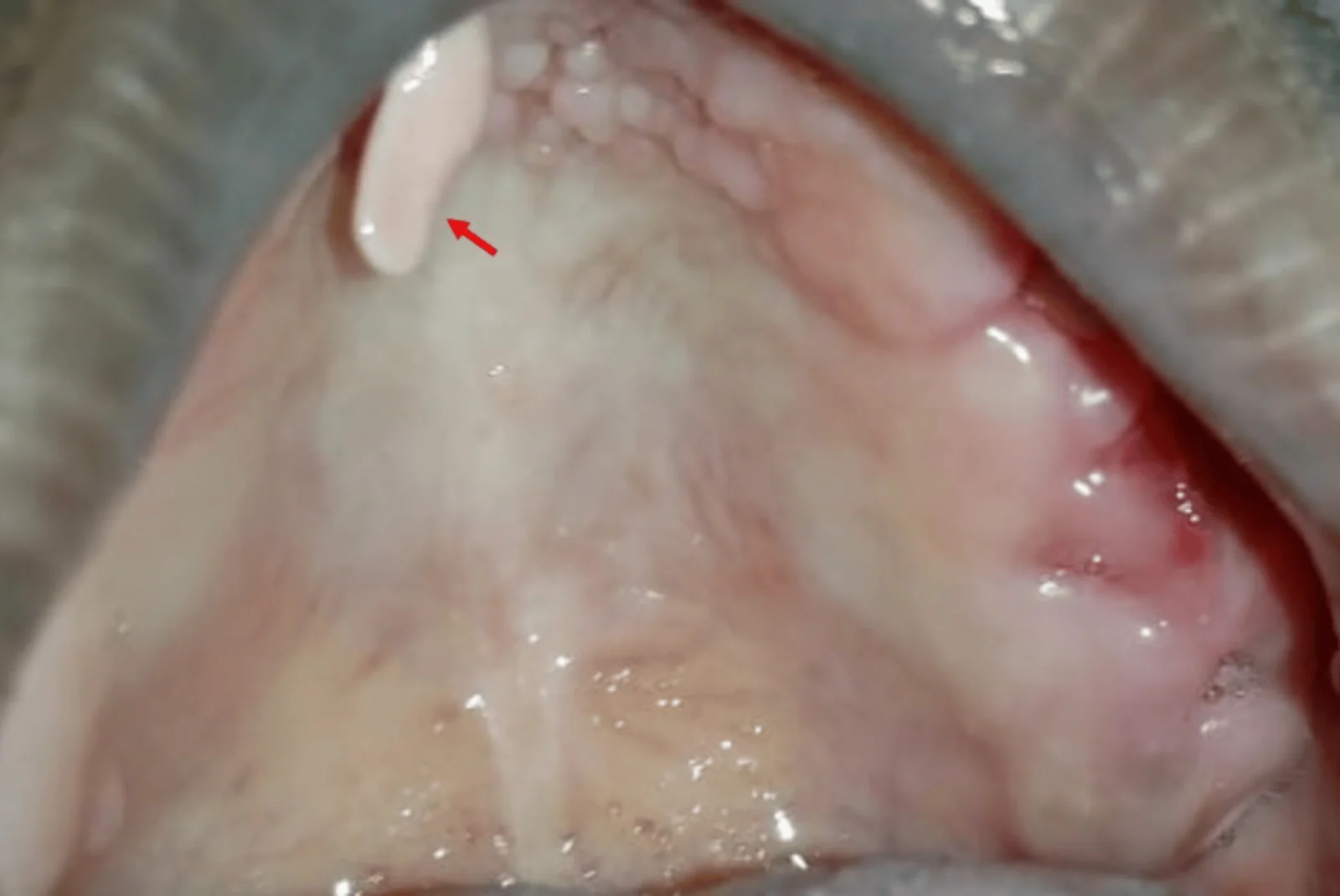
A close intraoral examination revealed a leaf-like growth with smooth margin hanging down in the mid-palatine raphe region on the hard palate (red arrow)

A provisional diagnosis of palatal papillomatosis was made based on the history of chronic denture-wearing and intraoral clinical findings. The differential diagnosis for papillary lesions of oral mucosa includes Cowden syndrome, squamous papilloma, verruca vulgaris, condyloma acuminatum, Schwarts-Burgess syndrome, stomatitis nicotina palati, denture epulis, and oral nodular chronic hyperplastic candidiasis. The patient was seronegative for HPV and HIV. Correlating the history of prolonged denture wearing and clinical findings, a final diagnosis of palatal papillomatosis was made.

Management 

The patient was referred to an oral maxillofacial surgeon for surgical ablation of growth on the hard palate mucosa. However, the patient refused treatment since it was painless and did not give consent for its surgical removal.

Clinical significance

A unique clinical finding of inflammatory papillary hyperplasia of the palate reported in this case report was the presence of mixed clinical features of a flat-leaf-like growth, which hangs down in the center interspersed with multiple coalesced pebble-like nodular growths of varied diameters in the periphery. This clinical appearance was not reported in any of the previous literature case reports.

Limitation

The patient refused treatment and neglected to turn up for further follow-up consultations. This patient did not undergo culture or biopsy, as well as specialized investigations such as real-time polymerase chain reaction to detect specific candidal species.

## Discussion

Definition

Inflammatory papillary hyperplasia is a type of denture stomatitis resulting from continuous wearing of dentures throughout the day and night, poor oral hygiene, mouth breathing, lack of proper maintenance of dentures, and ill-fitting dentures [1].

Synonyms

"Granular denture stomatitis," "Pseudo papillomatosis," "Newton's type III denture stomatitis," "Palatal granulations," and "Palatal papillomatosis" [1].

Incidence and prevalence

Gual-Vaques P et al. stated that 109 of 2669 denture wearers had inflammatory papillary hyperplasia, with a prevalence rate of about 4.43% [1]. Ettinger RL stated that there is a prevalence of 17% among 700 denture wearers [2]. Tucker KM and Heget HS stated that the incidence of palatal papillomatosis predominantly includes males 20-89 years of age screened among 892 dental patients in Veteran Administration Hospital, Oklahoma City, who wore the denture for more than 24 hours [3].

Etiology

The etiology of inflammatory papillary hyperplasia is multifactorial, which includes deflective occlusal contacts; unsatisfactory occlusion from disharmony between centric relation and occlusion, resulting in frictional or skidding or displacing movement of the denture bases; irritation of the minor salivary glands on the hard palate; mouth breathing; poor oral hygiene; malocclusion of the fabricated dentures; and night and day chronic continuous wearing of the dentures without proper cleaning of the denture that facilitates colonization of candida [4]. Relief areas beneath complete dentures, such as a large suction chamber in the form of a suction cup, aid in better retention of removable maxillary complete dentures and push the tissues inward that eventually become fibrosed. Long-term use of steroid inhalers in bronchial asthma favors candida colonization, systemic factors such as diabetes, and low serum vitamin D [5,6]. Papillary growths are also caused by HPV, which enters the oral squamous epithelium by endocytosis. HPV binds to a particular receptor protein on the keratinocyte membrane and is incorporated into the cell [7]. The virus sheds its protein covering, and the viral DNA may then reproduce itself using host cell DNA building blocks [7]. These viruses produce early gene E6 and E7 proteins, which can control the host cell cycle or have mitotic capabilities. E6 attaches to p53, a protein that inhibits cell division; once attached, it is destroyed, and the inhibition of keratinocyte mitosis is lost. Similarly, E7 binds a protein known as Rb, and a disrupted cell cycle leads to the growth of oral squamous epithelium [7].

Classification

In 1962, Newton classified denture hyperplasia based on the extent of inflammation as type I: localized inflammation representing pinpoint erythema on the hard palate, type II: generalized palatal erythema covering the denture-bearing area, and type III: inflammatory papillary hyperplasia, which results from an inflammatory epithelial response due to chronic low-grade trauma or irritation from poor oral hygiene or ill-fitting dentures resulting in pebble-like nodular growths secondary to colonization by Candida species. The modified Newton classification of inflammatory papillary hyperplasia is based on a 24-scoring system that divides the hard palate into four quadrants based on the presence of a slight or intense degree of erythema of the lesion and its extent of palatal mucosa per quadrant on the hard palate [8] (Figure 3).

**Figure 3:**
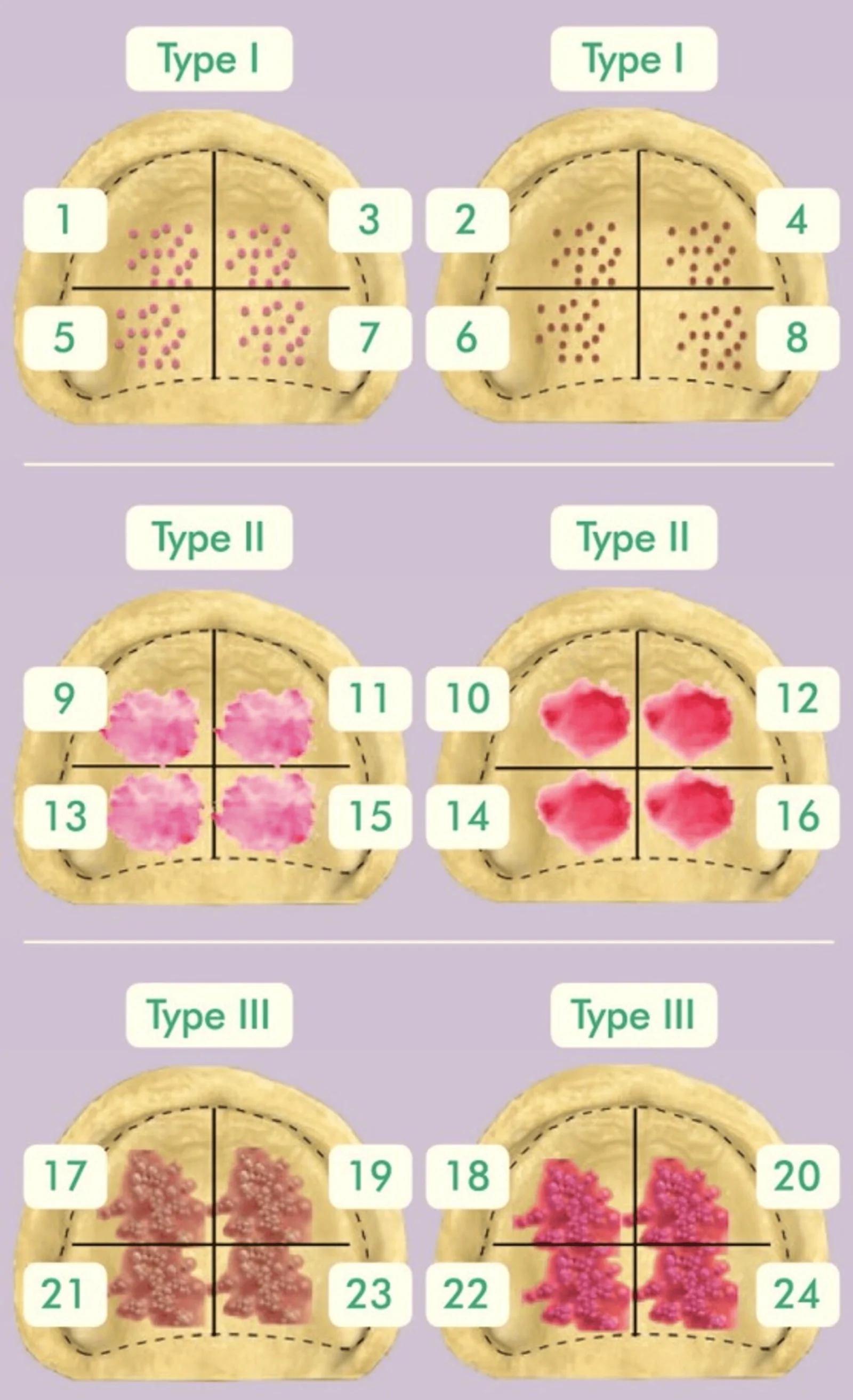
Modified Newton classification of inflammatory papillary hyperplasia Obtained permission from authors [8].

Clinical features 

Palatal papillomatosis is characterized by papilla-like projections of the palatal tissues [7]. These lesions are described as innumerable, concentric, coalesced papillomas, mulberrylike or sessile and lobulated, or consisting of multiple nodules, the same color as adjacent mucosa with a smooth or mossy surface to pebble-like with variations in the size of the projections, depth of the fissures, and degree of inflammation [7]. The condition is benign, but a burning sensation can sometimes develop due to colonization of candida organisms [7]. Females with lower serum Vitamin D levels were more prone to denture stomatitis aggravated by candidiasis due to the altered tissue response from postmenopausal hormonal changes [7]. Inflammatory papillary hyperplasia is a benign soft tissue reactive lesion to a local irritant that is usually associated with denture wear, soft and hard tissue deposits such as calculus, sharp tooth cusps, a broken filling, and other aggravating factors [8]. The lesion appears erythematous, fragile, and bleeds easily in the early stages. When fibrosis occurs, it takes on a hard, pink normochromic look, as in the current example [8]. Oral mucosal lesions of inflammatory papillary hyperplasia clinically mimic other epithelial hyperplasias such as focal epithelial hyperplasia, squamous papilloma, Cowden syndrome, denture epulis, and chronic nodular hyperplastic candidiasis. 

Differential diagnosis

Differential diagnoses include squamous papilloma, verruca vulgaris, Cowden syndrome, Schwartz-Burgess syndrome, florid papillomatosis, stomatitis nicotina palati, and oral nodular candidiasis.

Cowden syndrome is characterized by multiple papillomatous lesions involving gingiva, oral candidiasis, glossopyrosis, and multiple papillomatous lesions with a cobblestone or folded appearance on mucosa covering the alveolar ridge, tongue, buccal mucosa, squamous papilloma, and verruca vulgaris. Squamous papilloma usually has a cauliflower-like appearance on the hard palate mucosa, soft palate, tongue, and lips. Verruca vulgaris, or common warts caused by papillomavirus, clinically appear as multiple red or white papillary projections confined to any part of the skin, alveolar mucosa, and gingiva. Condyloma acuminatum appears sessile, with pink exophytic growths on the labial mucosa, lingual frenum, and soft palate. Multiple wart-like lesions characterized by florid cutaneous papillomatosis or Schwartz-Burgess syndrome usually occur on the skin, and those of oral florid papillomatosis occur on the lip and buccal mucosa. Stomatitis nicotina palati is characterized by reddish pinpoint areas on the center of raised multiple nodular lesions of the hard palate, usually in chronic smokers. Such reddish-pinpoint areas on the center of nodules are not seen in inflammatory papillary hyperplasia. In denture epulis, fibrous growths occur along the mucobuccal vestibule in the location of the denture flanges. Oral nodular chronic hyperplastic candidiasis occurs as multiple discrete papular nodular growths, usually on the tongue [9,10,11].

Scanning electron microscopy

Scanning electron microscopy revealed a predominant chronic inflammatory reaction with multiple tufted connective tissue papillary patterns and partly atrophic areas of oral epithelium in inflammatory papillary hyperplasia [12].

Treatment

The treatment modalities for inflammatory papillary hyperplasia vary depending on its duration and clinical appearance. Early or initial lesions with only erythematous, pinpoint, or multiple tiny granular appearances of 0.5 mm respond to non-surgical topical pharmacological therapies like antibacterial mouth rinses (0.12% chlorhexidine), nystatin 50,000/100,000 IU tablets used as lozenges, 2% miconazole nitrate gel, or systemic antifungal therapy such as fluconazole 200 mg per day for four weeks. The chronic or advanced stages of inflammatory papillary hyperplasia with the clinical appearance of reactive nodular or leaf-like soft tissue growths are managed by surgical modalities such as supraperiosteal stripping by blade-loop or bur with handpiece technique, lasers, or selective cryoablation. The photodynamic therapy uses a photosensitizing agent, PHOTOGEM®500 mg/mL, sprayed on the dentures and affected tissues of the hard palate [13]. After half an hour of incubation in the stored bag, the dentures are irradiated for 20 minutes at 37.5 J/cm^2^ by a source of light-emitting diode (LED) emitted by an LED photocuring unit, which activates photosensitizer at 455 nm, and the hard palate illuminated by LED for 20 minutes at 122 J/cm^2^ [13]. Photodynamic therapy is recommended thrice daily for 15 days to treat inflammatory papillary hyperplasia of the palate [13]. The other treatment modalities include the use of low-level laser therapy such as diode lasers, 0.12% chlorhexidine mouth rinse, 0.5% sodium hypochlorite, 500,000 IU of nystatin in the form of pastilles, lozenges to help disinfect tissue conditioners and denture reliners from colonization and growth of candidiasis, and disinfection of dentures by microwave [14]. A thin tinfoil (0.001 inch) is burnished in a hard palate impression on the cast before packing during denture processing [15]. This modification step during the fabrication of a removable maxillary complete denture aids in smoothing out the sharp edges of the denture, which prevents irritation and the formation of a hyperplastic tissue reaction on the oral mucosa [16]. Other treatment modalities involve selective cryotherapy resulting in cryoablation of affected tissues using a frozen cryoprobe, which results in the thawing of tissues [17]. Supraperiosteal excision is done using a blade-loop technique in which a blade-loop knife has a razor-like cutting blade, or the Paquette knife handle bowed blade concept pioneered by Uohara-Federbusch, which uses a bent one-half of a razor blade for cutting the palatal tissues [18]. A CO_2_ laser is followed by the fabrication of implant-supported prostheses in which crowns are retained by screws, which can be dismantled when necessary and make them less vulnerable to Candida colonization [19]. Bur with handpiece technique is used for the treatment of inflammatory papillary hyperplasia in which a surgical bur (Dura-Last, 3/32" Shank, Diatech) with a low-speed handpiece (Kavo micromotor Intramatic 181 M) at 20.000 rpm was employed to strip the palatal hyperplasic tissue from the palate [20].

## Conclusions

The varied clinical appearance of inflammatory palatal papillary hyperplasia as soft tissue overgrowth with multiple coalesced nodules, pebbles, mulberry, and grape or leaf-like clusters on the hard palate mucosa remains a diagnostic dilemma and challenge to dentists or oral physicians in diagnosing this condition. Such lesions can be mistaken for oral cancer or viral infections like HIV and HPV. It is prudential to screen patients with inflammatory papillary hyperplasia for HIV and HPV infections. Topical therapies using four-week antibacterial mouth rinses, nystatin tablets as lozenges, or antifungal therapy with fluconazole can resolve smaller-sized nodular lesions less than 1 mm in diameter. Larger lesions greater than 2 mm in diameter do not resolve by topical antibacterial or antifungal therapy and require surgical ablation by either subperiosteal excision, Co_2_ laser or cryotherapy, or screw-retained implant prosthesis, which are dismantled easily during the period of plaque buildup around the denture, aid in the maintenance of proper hygiene, and prevent them from turning to deep periodontal pockets that favor the growth of candidiasis. Oral physicians must instruct and motivate such patients to periodically check for any progression of such lesions. Periodic checkups with oral physicians and the disinfection of dentures can help alleviate this benign condition, which can induce fear of cancerophobia and anxiety in patients. Proper oral and denture hygiene maintenance helps resolve this condition early and prevent invasive surgical procedures.
